# Extensive multiregional urea elevations in a case-control study of vascular dementia point toward a novel shared mechanism of disease amongst the age-related dementias

**DOI:** 10.3389/fnmol.2023.1215637

**Published:** 2023-07-13

**Authors:** Sasha A. Philbert, Jingshu Xu, Melissa Scholefield, Stefano Patassini, Stephanie J. Church, Richard D. Unwin, Federico Roncaroli, Garth J. S. Cooper

**Affiliations:** ^1^Division of Cardiovascular Sciences, Faculty of Biology, Medicine and Health, School of Medical Sciences, Centre for Advanced Discovery and Experimental Therapeutics, The University of Manchester, Manchester Academic Health Science Centre, Manchester, United Kingdom; ^2^Faculty of Science, School of Biological Sciences, The University of Auckland, Auckland, New Zealand; ^3^Division of Neuroscience and Experimental Psychology, Faculty of Biology, Medicine and Health, School of Biology, Geoffrey Jefferson Brain Research Centre, The University of Manchester, Manchester, United Kingdom

**Keywords:** vascular dementia, age-related neurodegeneration, urea, metabolomics, mass spectrometry, dementia

## Abstract

**Introduction:**

Vascular dementia (VaD) is one of the most common causes of dementia among the elderly. Despite this, the molecular basis of VaD remains poorly characterized when compared to other age-related dementias. Pervasive cerebral elevations of urea have recently been reported in several dementias; however, a similar analysis was not yet available for VaD.

**Methods:**

Here, we utilized ultra-high-performance liquid chromatography-tandem mass spectrometry (UHPLC-MS/MS) to measure urea levels from seven brain regions in post-mortem tissue from cases of VaD (*n* = 10) and controls (n = 8/9). Brain-urea measurements from our previous investigations of several dementias were also used to generate comparisons with VaD.

**Results:**

Elevated urea levels ranging from 2.2- to 2.4-fold-change in VaD cases were identified in six out of the seven regions analysed, which are similar in magnitude to those observed in uremic encephalopathy. Fold-elevation of urea was highest in the basal ganglia and hippocampus (2.4-fold-change), consistent with the observation that these regions are severely affected in VaD.

**Discussion:**

Taken together, these data not only describe a multiregional elevation of brain-urea levels in VaD but also imply the existence of a common urea-mediated disease mechanism that is now known to be present in at least four of the main age-related dementias.

## 1. Introduction

Vascular dementia (VaD) is broadly characterized as the second most common form of age-related dementia, after Alzheimer’s disease (AD), with VaD prevalence rates in Europe ranging from 2.2 to 16.3% between the ages of 65–85+ years (Fratiglioni et al., 2000). VaD is defined as a heterogeneous class of brain disorders that develop from global or focal effects of cerebrovascular disease, which can subsequently lead to or cause cognitive impairment or dementia, behavioral abnormalities, and motor dysfunction (Looi and Sachdev, 1999; Bazner et al., 2000; Sachdev et al., 2004). The most common cerebrovascular pathologies in VaD are small vessel disease secondary to arteriosclerosis and amyloid angiopathy (Deramecourt et al., 2012). Concomitant small vessel disease is often present with AD pathology; thus, highlighting the high degree of pathogenic overlap between the two diseases (Deramecourt et al., 2012; Attems and Jellinger, 2014). There are currently no therapeutic options that yield significant disease-modifying effects in VaD and available therapies are largely restricted to the repurposing of medications used to treat AD (Sun, 2018). Although considerable advances have been made regarding our understanding of downstream pathophysiological processes in VaD, it remains to be determined what the precise molecular events underlying the disease are.

**Figure 1:**
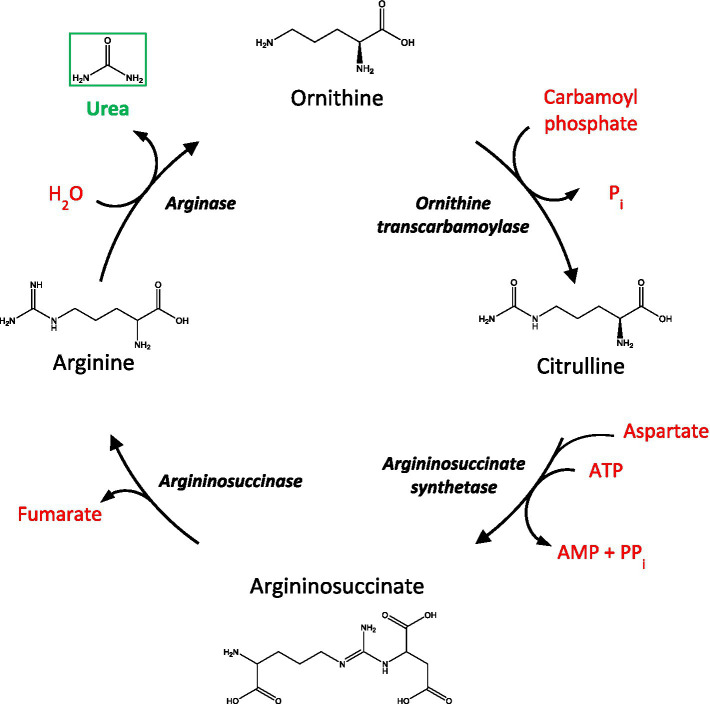
A schematic of the urea cycle. AMP, adenosine monophosphate; ATP, adenosine triphosphate; Pi, inorganic phosphate, PPi, inorganic pyrophosphate.

The urea cycle functions to convert toxic nitrogenous waste products, formed after protein catabolism, to urea before being excreted by the kidneys into the urine (Figure 1). Although urea is commonly regarded as a ‘less toxic’ derivative of ammonia, high levels of urea can also exert deleterious effects at the cellular and systemic level (Lau and Vaziri, 2017). Interestingly, mounting evidence now proposes the potential involvement of urea toxicity in various age-related dementias, including AD (Xu et al., 2016), Huntington’s disease (HD; Patassini et al., 2015; Handley et al., 2017), and Parkinson’s disease dementia (PDD) (Scholefield et al., 2021). For AD, in particular, recent evidence suggests that a fully functioning urea cycle is present in astrocytes, which is suggested to be implicated in amyloid beta clearance (Ju et al., 2022). Likewise, the upregulation of arginase, the enzyme responsible for catalyzing the hydrolysis of arginine to ornithine and urea, has also been observed in the brains of AD patients (Colton et al., 2006) and AD animal models (Polis et al., 2018). Nevertheless, despite these findings, there are currently no reports from studies that have investigated urea levels in VaD brain tissue. Some studies have identified systemic urea imbalances in patients with ischemic stroke (Schrock et al., 2012; Bhatia et al., 2015) and cardiovascular disease (Lan et al., 2021), which are associated with VaD. Although, while helpful, these studies do not explore the direct mechanisms that lead to altered cerebral urea metabolism in VaD. To ascertain whether cerebral urea concentrations are elevated in cases with small vessel disease-causing VaD, to levels previously observed in our group’s investigations of AD, HD, and PDD, we used ultra-high-performance liquid chromatography-tandem mass spectrometry (UHPLC-MS/MS) to quantify urea levels in human post-mortem tissue in seven brain regions from VaD cases (*n =* 10) and controls (*n =* 8/9) with similar age and post-mortem-delay (PMD) values. Our research hypothesis was that widespread elevated urea levels would be evident in VaD in brains comparable to those observed in our previous investigations of age-related dementias. Here, we observed widespread urea elevations in six out of the seven regions analysed. Although not as high as previously reported in other age-related dementias, the urea concentrations seen in VaD suggest a novel mechanism of toxicity via elevated urea levels, similar to those seen in uremic encephalopathy.

## 2. Methods

### 2.1. Human ethics

All experiments were performed in accordance with relevant UK and international guidelines and regulations as stated below. The study of post-mortem VaD/control tissue received local Research Ethics Committee approval (18/SW/0029) supplied by the South West Dementia Brain Bank (SWDBB). Informed consent for the collection of tissue was obtained by the SWDBB. Consents for the collection of AD (Xu et al., 2016), HD (Patassini et al., 2015), and PDD (Scholefield et al., 2021) tissue were as previously stated.

### 2.2. Case selection

Brain tissues from the 10 VaD cases, all of whom had a confirmed neuropathological diagnosis of small vessel disease at post-mortem examination and 10 controls with similar age, sex, and PMD values were obtained from the SWDBB. Although no statistical packages/models were used for the matching of cases and controls in this study, we acknowledged the *a priori*-agreed objective to obtain cases and controls that were not significantly different for sex, age, or PMD. A sample size of 20 (10 VaD and 10 control samples) was chosen based on the adequacy of data from our group’s previous investigations of post-mortem urea levels in various age-related dementias with similar sample sizes (Patassini et al., 2015; Xu et al., 2016; Scholefield et al., 2021). Sets of tissue from the thalamus (TH), basal ganglia (BG), cingulate gyrus (CG), frontal gyrus (FG), middle temporal gyrus (MTG), occipital cortex (OC), and hippocampus (HP) were acquired. These regions were selected on the basis that they are commonly affected in VaD, as well as allowing for a direct comparison to be made with our group’s previous studies involving brain-urea quantification in AD, HD, and PDD (Patassini et al., 2015; Xu et al., 2016; Scholefield et al., 2021). Although cerebrovascular damage is also evident in the hindbrain of VaD patients, analysis of hindbrain tissue in the present study was not performed due to the insufficient availability of tissue. Mean PMD was ≤38 h for VaD cases and controls; importantly, extended post-mortem delay of up to 72 h has been shown not to interfere with brain-urea levels (Scholefield et al., 2020). The sampling of post-mortem VaD tissue from multiple regions was chosen to enable both the identification of inter-regional differences in VaD and to ensure informative comparisons with brain-urea measurements from our prior datasets from AD, HD, and PDD.

The inclusion criteria employed for VaD cases included: confirmed neuropathological diagnosis of VaD at post-mortem examination; AD Braak stage of 3 or less (although only case 1105 had a Braak stage of 3); non-amyloid small vessel disease; none-to-moderate cerebral amyloid angiopathy; histopathological evidence of microinfarction; and no histopathological evidence of other neurological diseases likely to cause dementia. Controls had no clinical history of dementia and no other neurological abnormalities.

The Montreal Cognitive Assessment (MoCA), which is currently said to be the best neuropsychometric test for the assessment of VaD (Freitas et al., 2012), was recorded in eight of the 10 VaD cases. It should also be noted that the cause of death for patient 1008 was listed as VaD and type 2 diabetes (T2D) (Supplementary Table S1). Although T2D is a prominent risk factor for VaD, it can also itself lead directly to dementia or cognitive impairment (Xue et al., 2019).

Urea levels from the HP in the present study were generated using tissue that was initially intended for a preliminary single-region investigation of urea levels in VaD post-mortem tissue. However, due to the limited tissue availability, the same VaD cohort that was used for our group’s investigation of VaD hippocampal tissue (Supplementary Table S1; Philbert et al., 2022) could not be obtained for the remaining regions that were analysed in this study. Group characteristics for VaD cases and controls were as shown in Table 1 (excluding the hippocampal cohort from the preliminary investigation) and individual patient characteristics, including age and PMD in Supplementary Tables S1, S2. Group characteristics for the hippocampal cohort are shown in Table 2.

**Table 1:** Group characteristics excluding the hippocampal cohort.

Variable	Control	VaD
Number	9	10
Age	82 (69–94)	84 (72–98)
Male sex, *n* (%)	3 (30)	4 (40)
*Post-mortem* delay (h)	36 (24–44.25)	34 (20–54)
Brain weight (g)	1,197 (1,032–1,345)	1,220 (1,060–1,460)
Water content (%)	81.3 (79.8–83.8)	81.4 (77.5–84.2)
Wet-wt/dry-wt	5.57 (4.94–6.23)	5.67 (4.91–6.42)

Values are age, post-mortem delay, brain weight, and water content, mean (range); wet-wt/dry-wt ratio, mean (±95% CI) averaged across all samples. After the control with renal failure was removed, the cohort no longer contained the same number of males per group. All other differences were non-significant.

**Table 2:** Hippocampal cohort group characteristics.

Variable	Control	VaD
Number	8	10
Age	82 (69–94)	84 (72–98)
Male sex, *n* (%)	2 (20)	4 (40)
*Post-mortem* delay (h)	38 (29.5–44.25)	35 (20–54)
Brain weight (g)	1,201 (1032–1,345)	1,211 (1060–1,460)
Water content (%)	82.2 (78.7–85.5)	82.8 (78.7–86.3)
Wet-wt/dry-wt	5.71 (5.10–6.32)	5.94 (5.32–6.56)

Values are age, post-mortem delay, brain weight and water content, mean (range); wet-wt/dry-wt ratio, mean (±95% CI) averaged across all samples. After the controls with renal failure were removed, the cohort no longer contained the same number of males per group. All other differences were non-significant.

One control (**sample 781**) from the VaD study was excluded due to the presence of concomitant acute renal failure, which can cause uremic encephalopathy via elevated systemic urea levels. A second control (**sample 122**) was excluded from the HP analysis only, due to the presence of a urinary tract infection with elevated blood-urea levels at the time of death, consistent with potentially undiagnosed renal failure.

### 2.3. Tissue dissection

All samples were transported from the SWDBB to our University of Manchester laboratory on dry ice and then stored at −80°C. Samples were thawed briefly on ice before being cut into 20 mg aliquots (±2 mg; wet weight) using a ceramic scalpel and placed into 2 mL “Safe-Lock” microcentrifuge Eppendorf tubes (Eppendorf AG; Hamburg, Germany). Samples were stored at −80°C pending tissue extraction.

### 2.4. Tissue extraction

All brain samples were extracted in 800 μL 50:50 (v/v) methanol:chloroform mixture, which contained a stable isotopically-labeled urea internal standard (98 atom % ^15^N, 99% (CP); 316,830 Sigma–Aldrich) with a final concentration of 1 mM labeled urea in the extraction solvent (maintained at −20°C for a minimum of 4 h). Samples were placed in a TissueLyser plate (stored at −80°C for an hour before use) and extracted at 25 Hz for 10 min with a single 3 mm-tungsten carbide bead per sample in a TissueLyser bead homogeniser (Qiagen, Manchester, UK). After extraction, 400 mL of LC–MS grade water was added to each sample before it was briefly vortexed and then centrifuged at 2,400 × g for 10 min to ensure phase separation. 200 μL of the polar methanol phase from each sample was then transferred to a new pre-labeled microcentrifuge tube and dried overnight using a centrifugal concentrator (Savant SpeedVac^™^; Thermo-Fisher, Waltham, MA).

Once dried, 400 μL of 0.1% formic acid was added to each sample before it was briefly vortexed. Then, 100 μL of the resulting solution was transferred to 300 μL-insert autosampler vials, along with two extraction blanks containing 100 μL 0.1% v/v formic acid only. To generate the standard curve, 10 μL of the labeled urea internal standard together with an unlabeled external urea standard (urea analytical standard, 56180 Supelco, PA, United States) were added to 0.1% formic acid to achieve a final volume of 200 μL per standard, containing concentrations of 0–5,000 μL unlabeled urea in 0.1% v/v formic acid. Three quality control (QC) samples were also prepared containing 10 μL labeled urea and 20, 200, and 2,000 μL of unlabeled urea in 0.1% v/v formic acid.

### 2.5. Ultra-high-performance liquid chromatography-tandem mass spectrometry

Cerebral post-mortem urea levels were quantified via ultra-high-performance liquid chromatography-tandem mass spectrometry (UHPLC-MS/MS) using a TSQ Vantage triple quadrupole mass spectrometer coupled with an Accela UHPLC system (Thermo Fisher Scientific, MA, United States). Separation was carried out on a Hypersil Gold AQ column with a diameter of 2.1 mm, length of 100 mm, and particle size of 1.9 mm (ThermoFisher Scientific) maintained at 25°C with a 0.5 mm pre-column filter (Thermo Fisher Scientific). Gradient elution was performed using 0.1% formic acid in water (A) and 0.1% formic acid in acetonitrile (B) at 300 mL/min. Urea and labeled urea internal standard were detected using electrospray ionization in positive ionization mode.

### 2.6. Data analysis

UHPLC-MS/MS data were analysed using LCQuan software (Thermo Fisher Scientific, MA, United States). Chromatographic peaks were identified based on expected retention time (RT) and compared against labeled urea internal standard peak RTs for each individual QC/standard/sample. Each peak was manually checked for correct identification. Standards and QC samples were excluded from analysis if the percentage difference from the calibration curve was >15% (or 20% for the lowest standard or QC sample). At least two out of the three QC samples for each brain region had a percentage difference below this threshold.

Quantification of urea in samples was performed using the ratio of urea peak area to internal standard peak area and comparison to the standard curve. Brain-urea concentrations were first exported to Excel and then corrected for sample weight and dilution, and finally converted to units of mmol/kg.

The significance of brain-urea case–control differences was determined using either Welch’s *t*-tests or Mann–Whitney U tests depending on whether or not data were found to be normally distributed using the Shapiro–Wilks test of normality. Multi-dementia case-variable differences were determined using the Brown–Forsythe and analysis of variance (ANOVA) test. Statistical calculations were performed using GraphPad Prism v9.2.0 (GraphPad; La Jolla, CA). *p-*values < 0.05 were considered significant and *p*-values < 0.10 have also been tabulated. *Post-hoc* statistical power and sample-size estimates were calculated using G*Power v3.1.9.4 (Faul et al., 2007).

### 2.7. Sensitivity analysis

Given the relatively small sample size used in the present study (*n* = 18/19), sensitivity analyses were employed to further understand the strength of association to potential or uncontrolled confounding variables. Box plots were fitted using SPSS (version 28.0.1.0) to identify outliers in all urea datasets, and the impact of distributional assumptions was determined by comparing data from both parametric and non-parametric methods (Thabane et al., 2013).

The risk ratio (RR), also referred to as relative risk, is used to the compare risk of a “health event” between two distinct groups, in this case, controls vs. VaD samples. RR was calculated using the following formula:


(1) $$\mathrm{RR}=\left(\frac{a}{b}\right)/\left(\frac{c}{d}\right)$$

where *a* and *c* correspond to the number of cases and controls with urea levels >95% of the upper CI limit of the controls, and *b* and *d* correspond to the total number of cases and controls in the cohort, respectively.

The *E*-value can be interpreted as “the minimum strength of association, on the risk ratio scale, that an unmeasured confounder would need to have with both the treatment and the outcome to fully explain away a specific treatment-outcome association, conditional on the measured covariates” (VanderWeele and Ding, 2017). *E*-values were calculated using the following formula:


(2) E-value=RR+sqrt{RR×(RR−1)}

Although the *p-*value is necessary to confirm whether an effect is present, the effect size is key to understanding the magnitude of the difference between groups. Here, Glass’s delta was applied, rather than Cohens’s *d*, due to differences in standard deviations between cases and controls. Glass’s delta was calculated using the following formula:


(3) Glass'sΔ=M1−M2/σcontrol

where *M*_1_ is the mean case value, *M*_2_ is the mean control value, and σ_control_ is the standard deviation of the control group. Effect size values between 0.2 and 0.5 were considered small, values between 0.5 and 0.8 were considered medium size, values between 0.8 and 1.3 were considered large, and values >1.3 were considered very large.

### 2.8. STROBE statement

We completed the Strengthening the Reporting of Observational Studies in Epidemiology (STROBE) checklist to ensure that the reporting of the present data was in line with best-practice recommendations (von Elm et al., 2007).

## 3. Results

### 3.1. Case characteristics

Tissues were obtained from seven brain regions from each of 10 cases with cerebrovascular disease and 10 controls with similar age, sex, and PMD values. However, two controls from the HP and one control from the remaining brain regions were removed due to the presence of diagnosed (in one case) or probable (in the other) renal failure, which can cause elevations of systemic and cerebral urea levels. The removal of these two cases led to a greater number of males in the VaD group (*n =* 4) compared to controls (*n =* 3; HP: *n =* 2), thus, moderately unbalancing the study group. No significant case–control differences were present for post-mortem delay (PMD), age, brain weight, brain-water content, or dry-weight/wet-weight tissue ratios between all control and VaD brain-tissue samples used in this study (Tables 1, 2).

The causes of death for both VaD cases and controls are reported in Supplementary Tables S1, S2. The cause of death for five cases (32, 92, 131, 170, and 347) was not specified by the SWDBB, but all had diagnoses of VaD. As previously discussed, due to limited tissue availability, the same VaD cohort that was used for our group’s pilot investigation using HP tissue (Supplementary Table S1) could not be obtained for the remaining regions analysed in this study. Therefore, these cohort differences will need to be considered when comparing brain-urea concentrations.

Despite the considerable efforts made to eliminate all other possible causes of dementia amongst the hippocampal VaD group, the limited availability of hippocampal tissue precluded complete fulfillment of the specified exclusion criteria. One VaD case (case 1008) in the hippocampal cohort was diagnosed with T2D, which is suggested to independently influence the progression of dementia/cognitive impairment (Xue et al., 2019). However, case 1008 did not appear to generate any detectable outlying urea values in the hippocampal dataset or in our group’s previous metallomic investigation using the same tissue (Philbert et al., 2022).

### 3.2. Multiregional urea elevations in VaD brain tissue

**Figure 2:**
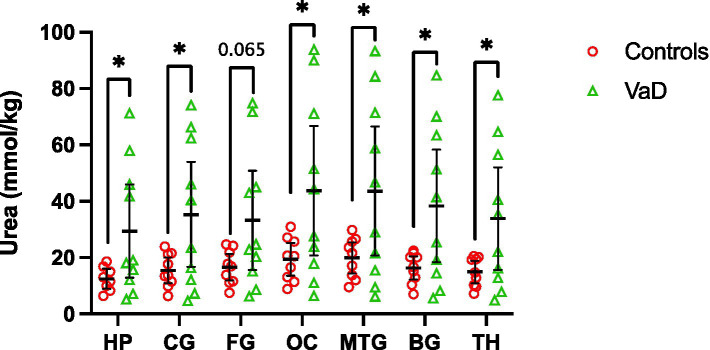
Multiregional urea concentrations in seven brain regions compared between control (red) and VaD (green) post-mortem tissue. Data are means ±95% CI. *p < 0.05. Non-standard abbreviations: TH, thalamus; BG, basal ganglia; CG, cingulate gyrus; FG, frontal gyrus; MTG, middle temporal gyrus; OC, occipital cortex; HP, hippocampus.

UHPLC-MS/MS analysis revealed statistically significant urea elevations in VaD in six out of the seven regions analysed (Table 3; Figure 2; Supplementary Table S3). Increased urea levels were apparent in the HP (*p* = 0.047), CG (*p* = 0.041), OC (*p* = 0.042), MTG (*p* = 0.046), BG (*p* = 0.035), and TH (*p* = 0.045) with the FG (*p* = 0.065) trending toward increased urea levels in VaD. Both the HP and BG exhibited the highest fold-change (FC) of 2.4 in cases compared to controls, with the remaining statistically significant regions ranging from 2.2 to 2.3 FC. No differences in inter-regional urea concentrations were detected for either cases or controls, suggesting a high degree of consistency amongst all regions.

**Table 3:** Multiregional urea concentrations in VaD and control brains.

Brain region	Control (mmol/kg)	VaD (mmol/kg)	Fold-change	*p*-value
Hippocampus	12.5 (9.0–16.0)	29.4 (12.9–45.9)	2.4	**0.047**
Cingulate gyrus	15.5 (10.9–20.1)	35.3 (16.7–53.9)	2.3	**0.041**
Frontal gyrus	16.6 (12.0–21.0)	33.3 (15.7–50.9)	2.0	0.065
Occipital cortex	19.4 (13.6–25.3)	43.8 (20.8–66.7)	2.3	**0.042**
Middle temporal gyrus	20.0 (14.5–25.6)	43.6 (20.8–66.5)	2.2	**0.046**
Basal ganglia	16.3 (12.2–20.4)	38.4 (18.4–58.4)	2.4	**0.035**
Thalamus	14.9 (11.0–18.9)	33.9 (15.7–52.0)	2.3	**0.045**
Grand-mean urea levels	16.5 (15.0–18.1)	36.8 (30.4–43.2)	2.2	**0.0001**

Data are means (±95% CI); *p*-values for significance of individual region between-group differences were calculated by Welch’s *t*-test based on urea measurements from control (*n* = 9/*n* = 8 for HP) and VaD (*n* = 10) brains. Grand-mean urea levels represent all urea concentrations from all brain regions used in this study. Case–control differences for grand-mean urea levels were determined by applying the Mann–Whitney U test (Con = 62, VaD = 70). Values in bold indicate statistically significant case-control differences.

Regional mean-urea concentrations were highest in the OC ([95% CI =20.8–66.7]; 43.76 mmol/kg) and lowest in HP ([95% CI = 12.9–45.9]; 29.41 mmol/kg) in cases, whereas, in controls, mean-urea concentrations were highest in the MTG ([95% CI = 14.5–25.6]; 19.98 mmol/kg) and lowest in the HP ([95% CI = 9.0–16.0]; 12.49 mmol/kg). Decreased HP urea concentrations for both cases and controls may be a product of cohort differences (as described in the Methods section), and, thus, must be considered when interpreting these results.

**Figure 3:**
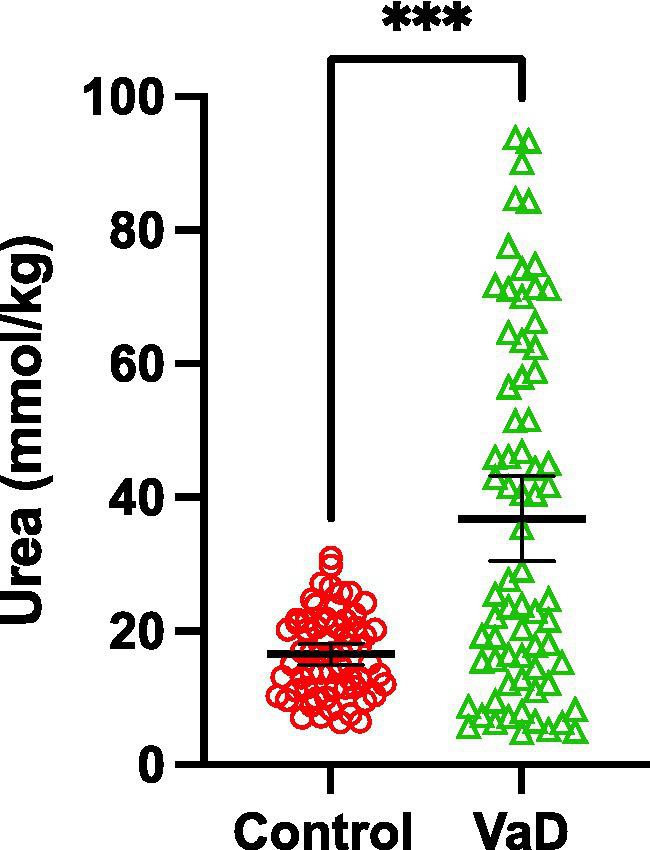
Grand-mean urea concentrations between control (red) and VaD (green) post-mortem tissue. Data are mean tissue urea concentrations ±95% CI from all brain regions investigated in this study. ***p < 0.0002.

Grand-mean analysis, using all urea measurements from all regions to determine global case-control differences (*n* = 132; Con *=* 62, VaD = 70), found mean-urea concentrations of 16.53 mmol/kg (95% CI = 15.0–18.1) in controls and 36.80 mmol/kg in cases ([95% CI = 30.4–43.2]; *p* = 0.0001; Table 3; Figure 3).

**Figure 4:**
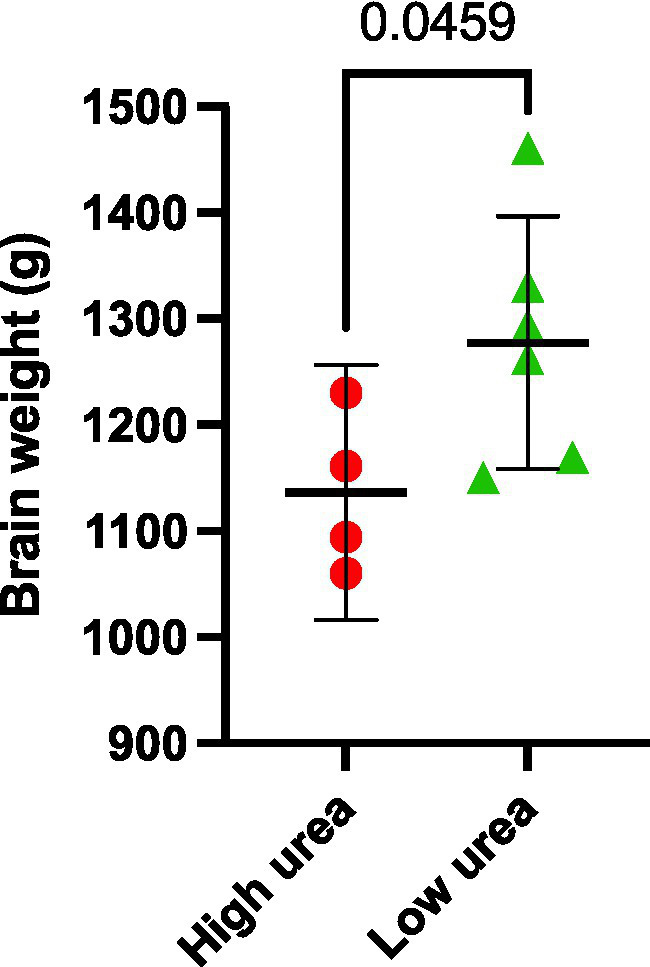
Post-mortem VaD brain weights. Data represents mean brain weights (wet weight) from VaD cases that were observed to have high or low brain-urea concentrations.

Interestingly, the same VaD cases (cases 32, 131, 347, and 787) consistently displayed higher multiregional urea values than the remaining cases in the VaD group, which were equivalent to that of the controls (Figure 2). Upon further inspection of the individual case characteristics (Supplementary Tables S1, S2), mean brain weights for these cases with consistently higher urea values were noticeably lower (1,136 g) compared to the remaining cases (1,277 g; Figure 4; *p* = 0.0459). No other differences in individual characteristics were evident in these cases.

**Figure 5:**
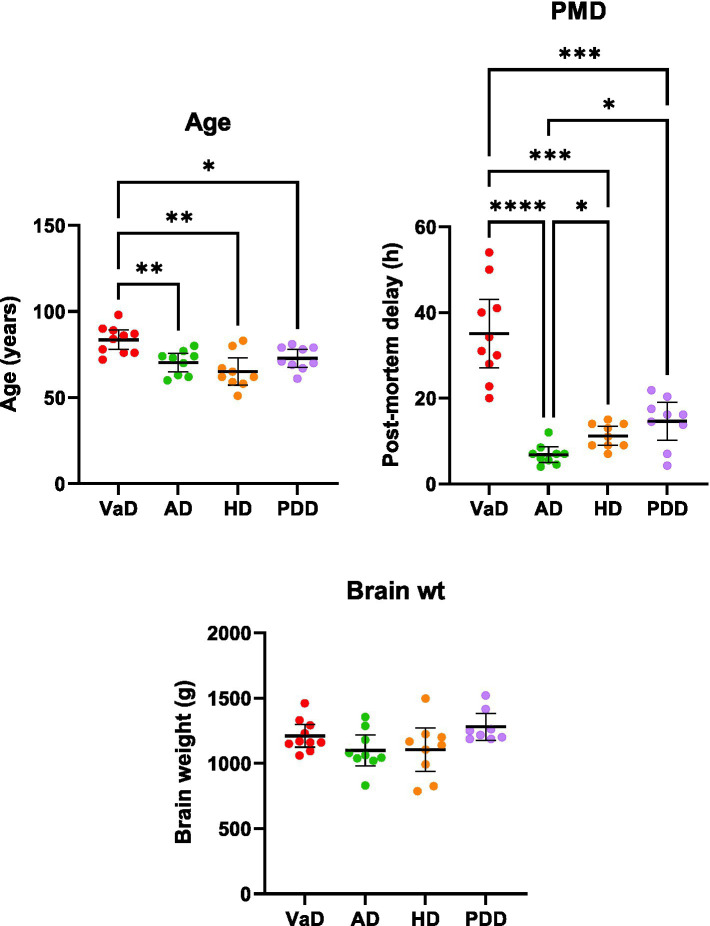
Multi-dementia case-variable comparisons. Data represents mean age, post-mortem delay, and brain weights (±95% CI) for VaD, AD, HD, and PDD cases. Brain weights are representative of wet tissue. Multi-dementia case variable differences were determined using Brown-Forsythe and ANOVA tests. ^*^p < 0.033, ^**^p < 0.0021, ^***^p < 0.0002, and ^****^p < 0.0001. Abbreviations: AD: Alzheimer’s disease; HD: Huntington’s disease; PDD: Parkinson’s disease dementia; PMD: Post-mortem delay; VaD: Vascular dementia.

Due to the relatively low sample sizes used in the present study (*n* = 18/19; Con = 8/9; VaD = 10), *post-hoc* power tests and sample size estimates were conducted to ensure the reliability of case–control differences. None of the regions analysed here met the sample size requirement according to regional sample estimates (*n =* ~52) and corresponding power analysis revealed statistical power levels ranging from 58 to 64%. Only the grand-mean urea analysis had a sample size greater than the required sample size estimate (*n =* 50), albeit with a statistical power of 60% (Supplementary Table S4).

### 3.3. VaD brain-urea comparisons with other dementias

The multiregional elevations of brain-urea concentrations in VaD described here were then compared with our group’s previous investigations of urea in AD (Xu et al., 2016), HD (Patassini et al., 2015), and PDD (Scholefield et al., 2021), wherein markedly increased urea levels were also apparent in all regions. Urea levels in VaD and PDD (*n* = 18; PDD = 9, Con = 9) were quantified using the same methodology (UHPLC–MS/MS), whereas urea levels in AD (*n* = 18; AD = 9, Con = 9) and HD (*n* = 18; HD = 9, Con = 9) were determined using gas chromatography–mass spectrometry. Multi-dementia case-variable comparisons revealed that VaD cases were significantly older than the other dementias analysed here (VaD vs. AD, *p* = 0.008; VaD vs. HD, *p* = 0.004; VaD vs. PDD, *p* = 0.031). In addition, multiple differences between dementia cases for mean PMDs were also observed (VaD vs. AD, *p* < 0.0001; VaD vs. HD, *p* = 0.0004; VaD vs. PDD, *p* = 0.0009; AD vs. HD, *p* = 0.017; AD vs. PDD, *p* = 0.017). However, no differences in brain weight were seen across all the dementias studied here (Supplementary Table S5; Figure 5).

A combined total of 16 brain regions were sampled across these four investigations; however, only three regions (HP, CG, and MTG) from AD, HD, and PDD overlapped with those used in the present study of VaD (Table 4). Therefore, a direct regional multi-dementia comparison of urea levels could only be achieved using these three brain regions. Of the age-related dementias, AD presented the most substantive overall increase in brain urea ([95% CI = 4.8–5.8]; 5.3 FC), followed by PDD ([95% CI = 3.9–4.6] 4.3 FC), HD ([95% CI = 3.2–3.6] 3.4 FC), and lastly VaD ([95% CI = 2.1–2.4] 2.2 FC; Table 4). The order of increased urea FC in these diseases remained the same even when the regional FC was restricted to just the HP, CG, and MTG (Table 4). Interestingly, severely affected brain regions, according to the pathogenesis of each respective disease, generally presented higher urea levels than the remaining regions.

**Table 4:** Brain urea fold-change comparisons between VaD and other age-related dementias.

Brain region	Fold-change in VaD (this study)	Fold-change in AD (Xu et al., 2016)	Fold-change in HD (Patassini et al., 2015)	Fold-change in PDD (Scholefield et al., 2021)
Hippocampus	2.4	6.5	3.6	4.2
Cingulate gyrus	2.3	5.3	3.5	5.5
Middle temporal gyrus	2.2	4.7	3.4	4.3
Occipital cortex	2.3	–	–	4.3
Frontal gyrus	2.0^†^	–	3.0	–
Basal ganglia	2.4	–	–	–
Thalamus	2.3	–	–	–
Sensory cortex	–	4.9	3.4	–
Cerebellum	–	4.9	3.6	3.7
Motor cortex	–	5.0	3.4	4.1
Substantia nigra	–	–	–	3.9
Medulla	–	–	–	4.6
Pons	–	–	–	3.9
Putamen	–	–	3.7	–
Globus pallidus	–	–	3.6	–
Entorhinal cortex	–	5.6	2.8	–
Overall	2.2 (2.1–2.4)	5.3 (4.8–5.8)	3.4 (3.2–3.6)	4.3 (3.9–4.6)
Overall disease comparison	2.3 (2.1–2.5)	5.5 (3.2–7.8)	3.5 (3.3–3.8)	4.6 (2.9–6.5)

Brain urea case-control fold-change comparisons between VaD (*n* = 10), AD (*n* = 9), HD (*n* = 9), and PDD (*n* = 9). Fold-change represents cases divided by the controls. Overall signifies the mean overall fold-change (±95% CI) for each investigation. Overall disease comparison signifies the mean overall fold-change (±95% CI) for all regions that were used for the multi-disease comparisons (highlighted in gray). Number of controls used in each cohort are as follows: VaD (*n* = 8/9), AD (*n* = 9), HD (*n* = 9), and PDD (*n* = 9). The order or urea fold-change across these dementias from highest to lowest is AD, PDD, HD, and lastly VaD.

† Only VaD urea levels in the FG were non-significant. All other values had value of *p*s < 0.05.

As T2D is a risk factor for both VaD and AD and can directly lead to dementia/cognitive impairment (Xue et al., 2019), urea levels were also quantified in the frontal cortex, temporal cortex, hippocampus, and meninges from cases with T2D and controls using the same UHPLC-MS/MS method described here, but no case–control differences in brain-urea levels were apparent (Supplementary Figure S1).

### 3.4. Sensitivity analysis

The fitting of box plots confirmed the absence of outliers in both cases (*n* = 10) and controls (*n* = 8/9) for all brain regions. However, while cases presented statistically significant increases in urea in six out of the seven regions using parametric methods (Welch’s *t*-test), such levels of significance were unable to be replicated using equivalent non-parametric methods (Mann–Whitney U test). Only grand-mean urea levels presented statistically significant elevations using both parametric (Welch’s *t*-test; *p* < 0.0001) and non-parametric methods (Mann–Whitney U; *p* = 0.0001).

For all regions, the RR and *E*-values ranged from 1.36 to 2.73 and 2.03 to 4.9, respectively (Supplementary Table S6). The effect sizes (measured by Glass’s Δ) for these regions were generally high, ranging between 2.73 and 4.12, with the BG showing the highest effect size (4.12) and the FG the lowest (2.73; Supplementary Table S6).

## 4. Discussion

Previously, our group has reported evidence of elevated cerebral urea levels in AD (Xu et al., 2016), HD (Patassini et al., 2015), and PDD (Scholefield et al., 2021) brain tissue, but to our knowledge, no such evidence existed for VaD prior to this study. In order to further understand the pattern of urea distribution in the age-related dementias, cerebral urea levels were quantified using equivalent mass-spectrometry-based methods in seven brain regions from 10 VaD cases and eight/nine controls, which identified widespread urea elevations in VaD cases. No differences were observed for age, post-mortem delay (PMD), brain weight, brain-water percentage, or wet-weight/dry-weight ratios between VaD cases and controls in the present study. As such, case-control differences in brain-urea levels are unlikely to be caused by these variables.

Following protein catabolism and the subsequent surplus of nitrogen-containing compounds, urea is produced through a series of steps, starting with the oxidative deamination of glutamate to form ammonium ion and α-ketoglutarate. It is usually said that these processes occur mainly in the liver. Excess ammonia is then metabolized via the urea cycle which catalyzes the formation of urea and is excreted into the urine via the kidneys. However, during periods of reduced kidney function, urea and other uremic toxins can accumulate which can lead to uremic encephalopathy, ensuing a cascade of downstream perturbations in the CNS, leading to memory loss, delusions, and seizures, among other symptoms (Rosner et al., 2022). In the present study, elevated urea levels ranging from 2.2 to 2.4 FC were identified in VaD post-mortem tissue in six out of the seven regions analysed. While some reports have shown associations between elevated blood-urea nitrogen/creatine ratios and ischemic stroke (Schrock et al., 2012; Bhatia et al., 2015), to the authors’ knowledge, this is the first study to provide direct evidence for elevated urea levels in the VaD brain. These data point toward a possible brain-specific mechanism that causes elevated cerebral urea levels, which will be further discussed in this section.

As there are currently no clinical methods that can quantify cerebral urea levels *in vivo*, the literature surrounding uremic encephalopathy has mainly focused on evidence derived from plasma creatinine and blood-urea nitrogen levels. Case reports of patients with uremic encephalopathy caused by renal failure showed an estimated 1.82–3.59 FC decrease in blood-urea nitrogen levels following dialysis and remission of symptoms related to uremic encephalopathy (Kim et al., 2016; Jia et al., 2017; Gong et al., 2018). Although these reports reflect systemic urea levels, and, thus, cannot be directly compared with corresponding measurements from the cerebral tissues, the case-control FC observed in the present study of VaD approximates those present in cases of uremic encephalopathy. Hence, this observation may imply that urea toxicity could play a mechanistic role in the pathophysiology of VaD.

As previously described, our group has also reported elevated urea levels in post-mortem brain tissue derived from cases of AD (Xu et al., 2016), HD (Patassini et al., 2015), and PDD (Scholefield et al., 2021), using equivalent mass-spectrometry-based techniques. After a comparison of all urea levels from the aforementioned dementia datasets, VaD displayed the lowest urea FC, followed by HD, PDD, and lastly AD. Although cerebral urea elevations may be multifactorial, one clear distinction between VaD and the other dementias presented here is the relative lack of neurodegenerative features (De Reuck et al., 2016). It is also important to note that T2D, which by itself is generally devoid of neurodegenerative pathology, did not show any case-control differences in urea. Thus, urea toxicity in the age-related dementias may be associated with neurodegenerative rather than cerebrovascular pathology, as commonly observed in VaD. However, cohort comparisons in the present study also revealed that VaD cases were on average older than those representing the other dementias, as well as there being widespread discrepancies among these dementias for PMD. While urea has been shown to remain stable in cerebral tissues for up to 72 h PMD (Scholefield et al., 2020), the effects of age in the VaD cases may have had an impact on the present results.

Interestingly, brain regions known to be severely affected in each respective dementia generally exhibited the highest urea FC. For example, the hippocampus and basal ganglia had the highest FC in VaD; the entorhinal cortex and hippocampus had the highest FC in AD; and the putamen had the highest FC in HD. However, this pattern of urea elevation did not extend to PDD, as the substantia nigra displayed a noticeably lower FC (3.9 FC) than the highest PDD regional FC (cingulate gyrus; 5.5 FC). Regardless, these data may imply that elevations of urea in the brain are associated, to a considerable extent, with the severity of regional pathology in age-related dementia. Taken together, the data shown here imply a shared urea-mediated mechanism amongst the age-related dementias that could potentially be used to inform novel therapies targeting all affected dementias.

The urea cycle contains four intermediates, namely citrulline, argininosuccinate, arginine, and ornithine. However, only a few studies have investigated these nitrogen-containing compounds in VaD. Mochizuki et al. (1996) reported elevated citrulline levels in the cerebrospinal fluid of cases with multi-infarct dementia, a subtype of VaD, but no differences in arginine, ornithine, or urea were observed. By contrast, Fleszar et al. (2019) noted decreased serum concentrations of citrulline and arginine in VaD patients. However, unlike other age-related dementias, several components of the urea cycle are still yet to be quantified in VaD, thus, making it hard to resolve the precise mechanism responsible for the urea phenotype observed here. Therefore, a thorough investigation of all urea-cycle intermediates and associated enzymes is needed to determine the role of the urea cycle more completely in VaD.

One hypothesis for the origin of elevated brain-urea levels in these dementias is an increase in protein catabolism in the brain as a result of underlying pathogenic processes. This hypothesis would certainly fit the data presented here as VaD cases with higher urea concentrations had considerably lower brain weights than cases with lower urea concentrations, possibly due to increases in protein catabolism and subsequent brain atrophy. While it is largely considered that the brain does not contain a complete urea cycle and, thus, cannot be responsible for elevations in urea, reports from other dementias that present equivalent cerebral urea phenotypes provide conflicting evidence. In an OVT73 sheep model of prodromal HD, which also displayed elevated cerebral urea, no significant differences or negligible expression of transcripts encoding key urea-cycle enzymes were observed in the striatum (Handley et al., 2017). In addition, decreased ornithine levels have been reported in HD human brain tissue (Patassini et al., 2016), thus, suggesting that elevated cerebral urea levels may not be a product of urea-cycle perturbations in the brain. Although there is limited evidence in PDD, one study showed decreased arginine levels, but no detection of other urea-cycle intermediates, as well as increased mRNA expression of arginase and argininosuccinate lyase in a drosophila model of Parkinson’s disease (Solana-Manrique et al., 2022). However, in AD, a complete astrocytic urea cycle has recently been reported, which is suggested to be implicated in amyloid-beta plaque clearance (Ju et al., 2022). Alterations in several other metabolites linked to the urea cycle and glutamate metabolism have also been observed in AD (Mahajan et al., 2020). In addition, ornithine transcarbamylase was reported to be elevated in the CSF of AD patients (Bensemain et al., 2009) and despite the detection of the remaining urea-cycle enzymes in AD brain tissue, only Arginase 2 was found to be increased (Hansmannel et al., 2010). Therefore, while it is still not clear whether the brain contains a complete urea cycle, reports of urea-cycle components in other dementias imply that a similar mechanism is likely causing the elevation in cerebral urea. However, what this mechanism might be will require further investigation.

As the urea cycle functions to detoxify ammonia in the form of urea, disturbances in the glutamate–glutamine cycle, the brain’s primary pathway against ammonia toxicity, could likely be a contributing factor to the elevated cerebral urea levels observed here in VaD. Glutamine is strictly synthesised in astrocytes by means of glutamine synthetase, which catalyses the ATP-dependent conversion on glutamate and ammonia to glutamine (Schousboe et al., 2014). While the glutamate–glutamine cycle cannot directly produce urea, altered cycle activity could further add to the possible surplus of ammonia levels in the brain, which could then influence urea levels via the urea cycle. Although this mechanism has yet to be studied in VaD brains, altered glutamate–glutamine homeostasis has been reported in several neurodegenerative diseases, including AD, HD, amyotrophic lateral sclerosis, and frontotemporal dementia (Aldana et al., 2020; Andersen and Schousboe, 2023). Glutamine synthesis is also directly linked to astrocyte energy metabolism, which has shown to be implicated in AD and HD (Boussicault et al., 2014; Ryu et al., 2021; Zysk et al., 2023). Therefore, while there is limited available data regarding the glutamate–glutamine cycle in VaD, given its ammonia-clearance capabilities and reported dysfunction in other neurodegenerative diseases, disruption to this cycle may operate in tandem with the urea cycle, or an additional mechanism, to cause the cerebral urea phenotype observed in this study. However, due to the lack of data in VaD, this is purely speculative.

The principal site of urea production in the body is the liver. As such, it is plausible that elevated urea levels in the brain originate from a systemic dysfunction of urea metabolism. Although the osmotic properties of urea mean that it is slow to cross brain capillaries (Sterns et al., 2015), disruption of the blood–brain barrier in cerebrovascular and neurodegenerative diseases could provide a more viable route for urea into the CNS (Xiao et al., 2020). However, the systemic elevation of urea, also referred to as uremia, is not known to develop in the age-related dementias, and uremia was not characterized by pathological examination in any of the samples used in this study. This suggests that elevations in cerebral urea levels are unlikely to be caused by uremia, thus, indicating that increased urea levels originate within the brain, albeit through a potentially different mechanism to that provided by a version of the urea cycle in the brain. However, as the absence of uremia is assumed, the question of why no case–control differences in brain-water percentage were apparent in these samples due to the osmotic effect of urea still remains (Tables 1, 2). One potential explanation might be that the osmotic gradient between the periphery and the CNS was not great enough to promote the net movement of water into the brain, although further research may be necessary to confirm this.

Even though elevated urea levels are assumed to originate in the brain, the issue of why excess urea is not being cleared from the CNS remains to be determined. There are two families of facilitative urea transporters known to exist in mammals, UT-A and UT-B, which are encoded by *SLC14A2* and *SLC14A1*, respectively (Sands, 2003). Whereas UT-A transporters are predominantly expressed in the kidney, UT-B transporters are shown to be expressed in a wide variety of organs, including the brain (Yu et al., 2019). Although studies of UT-B transporters in neurological diseases are lacking, our group has previously identified increased expression of *Slc14a1* in a transgenic sheep model of HD (Handley et al., 2017). UT-B knockout mouse models have also provided evidence for widespread increases in brain-urea levels (Li et al., 2012). Although a urea transporter deficit in the brain would seem the most likely answer to the sustained urea levels seen here, data from HD may imply that this is indeed not the case. Thus, consistent with our previous argument regarding *SLC14A1* in HD (Handley et al., 2017), UT-B transporters in VaD and other dementias are likely to be increased to compensate for the markedly elevated brain-urea levels. One possible explanation is that this compensatory mechanism is simply overwhelmed due to the profound increases in brain urea, approximately a 2.2–5.2 FC increase across all dementias (Table 4), which may explain the observed elevated brain-urea levels at post-mortem.

Sensitivity analyses were conducted to further understand the strength of association that potential or uncontrolled confounding variables had on the results of this study. Firstly, boxplots were used to exclude the presence of outliers that might have affected the interpretation of results. Secondly, case–control urea *p*-values obtained using parametric methods were compared with equivalent non-parametric methods to evaluate the robustness of the data. However, while only the grand-mean urea levels satisfied the criterion of *p* < 0.05 for both parametric and non-parametric methods, it is important to note that non-parametric methods have generally lower statistical power and that the present urea dataset was deemed to be normally distributed using the Shapiro–Wilk test of normality. Thirdly, the analysis of RRs and *E*-values presented ranges from 1.36 to 2.73 and 2.03 to 4.9, respectively. This means, if we were to take the lowest of these ranges (from the TH; RR = 1.36, *E*-value = 2.03), then the RR of 1.36 could explain away the effect (Glass’s Δ = 3.69) by an unmeasured confounder by a RR > 2.03; however, a RR < 2.03 could not do so. Therefore, it is plausible that a possible unmeasured confounder could have influenced the urea levels in some of the brain regions studied here. However, this is unlikely due to the uniformity of urea elevations in these VaD cases and the observed similar elevations of urea in our group’s prior investigations of dementia (Patassini et al., 2015; Xu et al., 2016; Scholefield et al., 2021).

Although this urea phenotype is now evident in four age-related dementias, including VaD, one clear weakness of the present study is the use of low sample sizes (Con: *n =* 8/9; VaD: *n =* 10). *Post-hoc* power tests and sample-size estimates were conducted to ensure the reliability of the observed case–control differences. However, only the grand-mean analysis satisfied the sample-estimate criteria and all differences had power levels <80%. Although the low power seen here is arguably due to the increased urea variability seen amongst the VaD cases, these data nonetheless highlight the need for larger cohort sizes for more robust measurements. Regarding the generalisability of this study, the samples used here were sourced from an established UK-based brain bank, which is seen to be representative of the general population. In addition, as urea metabolism is known to be tightly regulated by protein intake, urea levels could possibly be influenced by varying dietary control within patients and disease models (Young et al., 2000). Therefore, further analysis is needed, controlling for diet, to fully confirm the role of urea in VaD and other age-related dementias.

## 5. Conclusion

In summary, these data provide evidence for the widespread elevation of urea in VaD post-mortem brain tissue, which closely resembles that present in AD, HD, and PDD. Although the precise role of the urea cycle toward elevated cerebral urea levels remains to be determined, it is likely this urea phenotype originates in the brain, possibly via increased protein catabolism. Future studies should aim to uncover the precise molecular mechanism responsible for the elevation of cerebral urea in VaD, which could potentially be used to inform novel therapies targeting all affected age-related dementias.

## Data availability statement

The original contributions presented in the study are included in the article/Supplementary material, further inquiries can be directed to the corresponding author.

## Ethics statement

This study was conducted according to the guidelines of the Declaration of Helsinki, and received local Research Ethics Committee approval (18/SW/0029; 23 February 2018) supplied by the SWDBB, which is an NHS research ethics committee-approved tissue bank. Informed consent was obtained by the SWDBB from all individual participants that were included in the study. Consents for the collection of AD (9), HD (10), and PDD (12) tissue were as previously stated.

## Author contributions

SAP designed and performed experiments, analysed and interpreted data, and wrote the manuscript. JX, MS, and SP performed experiments and revised the manuscript. SC performed experiments and analysed the data. RU designed experiments, interpreted the data, and revised the manuscript. FR advised on the sampling of brain regions and revised the manuscript. GC designed the experiments, interpreted the data, revised the manuscript, and bears overall responsibility for the integrity of this manuscript and of the study. All authors contributed to the article and approved the submitted version.

## Funding

The authors acknowledge the following funding sources: the Endocore Research Trust (NZ) (60187); the Lee Trust (NZ); the Oakley Mental Health Research Foundation (NZ) (3456030; 3627092; 3701339; 3703253; 3702879); the Maurice and Phyllis Paykel Trust (3627036); the UKRI Medical Research Council (MR/N0284457/1); the NZ Ministry of Business, Innovation and Employment (PROP-57362-ENDRP-UOA; 3716382); The University of Manchester; the Northwest Regional Development Agency through a combined program grant to CADET; and facilitated by the Greater Manchester Comprehensive Local Research Network.

## Conflict of interest

The authors declare that the research was conducted in the absence of any commercial or financial relationships that could be construed as a potential conflict of interest.

## Publisher’s note

All claims expressed in this article are solely those of the authors and do not necessarily represent those of their affiliated organizations, or those of the publisher, the editors and the reviewers. Any product that may be evaluated in this article, or claim that may be made by its manufacturer, is not guaranteed or endorsed by the publisher.
