# Supplementary material for: Extensive multiregional urea elevations in a case-control study of vascular dementia point toward a novel shared mechanism of disease amongst the age-related dementias
Source: Front Mol Neurosci. 2023 Jul 13;16:1215637. doi: 10.3389/fnmol.2023.1215637 (PMC10372345; doi:10.3389/fnmol.2023.1215637)
Supplement: Supplementary file 1 [file Data_Sheet_1.docx]

Supplementary Material

| Table S1. Hippocampal tissue individual patient characteristics | | | | | | | |
| --- | --- | --- | --- | --- | --- | --- | --- |
| BBN Id | Code | Class | Age at death | Sex | Brain wt (g) | PMD (h) | Cause of death^*^ |
| BBN_8706 | 72 | Control | 72 | M | 1300 | 42 | Ruptured abdominal aortic aneurysm, ischaemic heart disease |
| BBN_8751 | 122^‡^ | Control | 82 | M | 1480 | 30 | Congestive cardiac failure, ischaemic heart disease, UTI |
| BBN_4205 | 781^‡^ | Control | 87 | M | 1364 | 24 | Acute renal failure, myeloma |
| BBN_19627 | 930 | Control | 94 | F | 1218 | 29.5 | Oesophageal carcinoma, ischemic heart disease |
| BBN_22625 | 943 | Control | 70 | F | 1032 | 33.25 | Carcinomatosis, metastatic squamous cell carcinoma of larynx |
| BBN_24311 | 948 | Control | 82 | F | 1135 | 36 | Carcinomatosis, ovarian/peritoneal carcinoma |
| BBN_24325 | 957 | Control | 86 | M | 1345 | 44.25 | Infective exacerbation of COPD |
| BBN006.30186 | 1067 | Control | 69 | F | 1121 | 38.25 | Metastatic pancreatic cancer |
| BBN006.31516 | 1083 | Control | 94 | F | 1166 | 43.25 | Pneumonia, frailty of old age |
| BBN006.32578 | 1092 | Control | 86 | F | 1288 | 36.25 | Mucinous adenocarcinoma of appendix |
| BBN_8667 | 32 | VaD | 84 | F | 1230 | 20 | Not specified^†^ |
| BBN_8724 | 92 | VaD | 72 | M | 1460 | 41 | Not specified^†^ |
| BBN_8760 | 131 | VaD | 86 | F | 1060 | 28 | Not specified^†^ |
| BBN_8799 | 170 | VaD | 90 | F | 1150 | 31 | Not specified^†^ |
| BBN_8861 | 232 | VaD | 89 | M | 1330 | 30 | Pulmonary embolism, ischaemic heart disease, old age |
| BBN_8975 | 347 | VaD | 76 | M | 1094 | 40 | Not specified^†^ |
| BBN_4208 | 787 | VaD | 78 | F | 1161 | 54 | Vascular dementia |
| BBN_19628 | 931 | VaD | 76 | F | 1293 | 50 | Urosepsis |
| BBN006.26572 | 1008 | VaD | 87 | M | 1160 | 34.25 | Old age, vascular dementia, leg ulcer, type-2 diabetes |
| BBN006.33638 | 1105 | VaD | 98 | F | 1169 | 22.75 | Vascular dementia |
| ^†^Those patients in the VaD Class whose cause of death was not specified in the database had VaD as determined by post-mortem examination but not specified at the time of writing as a cause of death in the SWDBB database. ^‡^Samples were excluded from analysis due to having concomitant disorders which can lead to elevated urea levels. ^*^Cause of death as specified by the SWDBB database. Abbreviations: BBN: Brain bank number; COPD: Chronic obstructive pulmonary disease; PMD: *Post-mortem* delay; UTI; Urinary tract infection; wt: Weight. | | | | | | | |

**Supplementary Tables**

| Table S2. SWDBB multiregional individual patient characteristics | | | | | | | |
| --- | --- | --- | --- | --- | --- | --- | --- |
| BBN Id | Code | Class | Age at death | Sex | Brain wt (g) | PMD (h) | Cause of Death^*^ |
| BBN_4205 | 781^‡^ | Control | 87 | M | 1364 | 24 | Acute renal failure, myeloma |
| BBN006.32578 | 1092 | Control | 86 | F | 1288 | 36.25 | Mucinous adenocarcinoma of appendix |
| BBN_9354 | 786 | Control | 85 | M | 1337 | 30.5 | Acute myocardial infarction due to ischaemic heart disease |
| BBN_19627 | 930 | Control | 94 | F | 1218 | 29.5 | Oesophageal carcinoma, ischaemic heart disease |
| BBN_22625 | 943 | Control | 70 | F | 1032 | 33.25 | Carcinomatosis, metastatic squamous cell carcinoma of larynx |
| BBN_24311 | 948 | Control | 82 | F | 1135 | 36 | Carcinomatosis, ovarian/peritoneal carcinoma |
| BBN_24312 | 949 | Control | 69 | M | 1132 | 31.25 | Non-small cell lung cancer |
| BBN_24325 | 957 | Control | 86 | M | 1345 | 44.25 | Infective exacerbation of COPD |
| BBN006.30186 | 1067 | Control | 69 | F | 1121 | 38.25 | Metastatic pancreatic cancer |
| BBN006.31516 | 1083 | Control | 94 | F | 1166 | 43.25 | Pneumonia, frailty of old age |
| BBN_8667 | 32 | VaD | 84 | F | 1230 | 20 | Not specified^†^ |
| BBN_8724 | 92 | VaD | 72 | M | 1460 | 20 | Not specified^†^ |
| BBN_8760 | 131 | VaD | 86 | F | 1060 | 28 | Not specified^†^ |
| BBN_8799 | 170 | VaD | 90 | F | 1150 | 31 | Not specified^†^ |
| BBN_8861 | 232 | VaD | 89 | M | 1330 | 30 | Pulmonary embolism, ischaemic heart disease, old age |
| BBN_8975 | 347 | VaD | 76 | M | 1094 | 40 | Not specified |
| BBN_9387 | 849 | VaD | 90 | M | 1262 | 45 | Dementia^†^ |
| BBN_19628 | 931 | VaD | 76 | F | 1293 | 50 | Urosepsis |
| BBN_4208 | 787 | VaD | 78 | F | 1161 | 54 | Vascular dementia |
| BBN006.32578 | 1105 | VaD | 98 | F | 1169 | 22.75 | Vascular dementia |
| ^†^Those patients in the VaD Class whose cause of death was not specified in the database had VaD as determined by post-mortem examination but not specified at the time of writing as a cause of death in the SWDBB database. ^‡^Sample 781 was excluded from analysis due to having acute renal failure which can lead to elevated urea levels. ^*^Cause of death as specified by the SWDBB database. Abbreviations: BBN: Brain bank number; COPD: Chronic obstructive pulmonary disease; PMD: *Post-mortem* delay; wt: Weight. | | | | | | | |

| **Table S3.** Raw multiregional urea concentrations in VaD and control post-mortem brain tissue | | | | | | | | | | |
| --- | --- | --- | --- | --- | --- | --- | --- | --- | --- | --- |
| Sample ID | | | Class | HP | CG | FG | OC | MTG | BG | TH |
| Hippocampus | All other brain regions | |  |  |  |  |  |  |  |  |
| **781** | 72 | | Control | 8.29 | **53.50** | **53.47** | **57.16** | **57.21** | **56.61** | **48.25** |
| 1092 | **122** | | Control | **33.56** | 21.53 | 24.20 | 27.13 | 26.60 | 20.23 | 20.19 |
| 786 | **781** | | Control | **39.87** | 23.95 | 24.74 | 30.98 | 29.78 | 22.42 | 19.27 |
| 930 | 930 | | Control | 11.53 | 13.42 | 13.86 | 16.52 | 17.10 | 15.24 | 12.91 |
| 943 | 943 | | Control | 15.02 | 17.68 | 17.76 | 20.73 | 23.61 | 20.11 | 19.48 |
| 948 | 948 | | Control | 6.49 | 6.41 | 7.57 | 8.84 | 9.54 | 7.06 | 7.24 |
| 949 | 957 | | Control | 13.08 | 9.89 | 11.66 | 11.43 | 12.05 | 10.38 | 9.73 |
| 957 | 1067 | | Control | 10.02 | 13.81 | 16.94 | 20.56 | 21.63 | 16.70 | 14.78 |
| 1067 | 1083 | | Control | 16.86 | 11.55 | 11.14 | 13.09 | 13.79 | 12.94 | 10.30 |
| 1083 | 1092 | | Control | 18.64 | 21.19 | 21.85 | 25.69 | 25.69 | 21.71 | 20.53 |
| 32 | 32 | | VaD | 45.93 | 62.38 | 71.70 | 71.12 | 71.37 | 63.41 | 56.50 |
| 92 | 92 | | VaD | 12.15 | 12.13 | 15.20 | 17.89 | 15.45 | 14.46 | 12.81 |
| 131 | 131 | | VaD | 41.74 | 46.08 | 45.00 | 51.51 | 58.77 | 51.40 | 40.51 |
| 170 | 170 | | VaD | 15.68 | 16.34 | 20.37 | 23.86 | 21.65 | 19.23 | 16.17 |
| 232 | 232 | | VaD | 7.08 | 7.30 | 8.59 | 11.05 | 9.47 | 8.22 | 7.83 |
| 347 | 347 | | VaD | 57.98 | 66.17 | 74.82 | 90.04 | 84.28 | 70.08 | 64.60 |
| 849 | 787 | | VaD | 71.22 | 40.49 | 42.97 | 44.32 | 46.79 | 41.51 | 35.34 |
| 931 | 931 | | VaD | 5.18 | 4.71 | 6.29 | 6.43 | 6.20 | 5.60 | 4.99 |
| 787 | 1008 | | VaD | 18.14 | 74.12 | 24.74 | 93.83 | 93.25 | 84.64 | 77.58 |
| 1105 | 1105 | | VaD | 19.02 | 23.56 | 22.84 | 27.59 | 29.07 | 25.43 | 22.14 |
|  | | Av. Control | | 12.49 | 15.49 | 16.64 | 19.44 | 19.98 | 16.31 | 14.94 |
|  | | Av. VaD | | 29.41 | 35.33 | 33.25 | 43.76 | 43.63 | 38.40 | 33.85 |
|  | | Fold-change | | 2.4 | 2.3 | 2.0 | 2.3 | 2.2 | 2.4 | 2.3 |
|  | | *p*-value | | 0.047 | 0.041 | 0.065 | 0.042 | 0.046 | 0.035 | 0.045 |
| Data are means (±95% CI); *p*-values for significance of individual region between-group differences were calculated by Welch’s *t*-test based on urea measurements from control (n = 9/n = 8 for HP) and VaD (n = 10) brains. Sample 781 and 122 (in bold) were removed due to the presence of concomitant renal failure which can affect urea levels. Abbreviations: BG: basal ganglia; CG: cingulate gyrus; FG: frontal gyrus; HP: hippocampus; MTG: middle temporal gyrus; OC: occipital cortex; TH: thalamus. | | | | | | | | | | |

| **Table S4**. Multiregional sample size and power estimates | | |
| --- | --- | --- |
| Brain region | Sample size estimates | Power estimates (%) |
| Hippocampus | 52 | 58 |
| Cingulate gyrus | 50 | 60 |
| Frontal gyrus | 62 | 50 |
| Occipital cortex | 50 | 60 |
| Middle temporal gyrus | 52 | 58 |
| Basal ganglia | 46 | 64 |
| Thalamus | 52 | 59 |
| Grand mean analysis | 50 | 60 |
| Required samples sizes were generated using a statistical power of 80% and an α error probability of 0.05. Post-hoc power analyses were generated using an α error probability of 0.05. Values were determined using G*Power (v. 3.1.9.4). | | |

| **Table S5.** Multi-dementia comparison cohort characteristics | | | | |
| --- | --- | --- | --- | --- |
| Variable | VaD | AD | HD | PDD |
| Number | 10 | 9 | 9 | 9 |
| Male sex, *n* (%) | 4 (40) | 4 (44) | 6 (66) | 5 (55.6) |
| Age | 84 (72-98) | 70 (60-80) | 65 (51-83) | 73 (61-81) |
| PMD (h) | 35 (20-54) | 6.8 (4-7) | 11.2 (7-15) | 14.6 (4.3-21.9) |
| Brain wt (g) | 1211 (1060-1460) | 1100 (831-1355) | 1104 (787-1497) | 1280 (1187-1520) |
| Data are means (range). *p*-values for the significance of between-groups differences were calculated by Brown-Forsythe and ANOVA test. For age, VaD was significantly different compared to all other dementias. For PMD, only the HD/PDD comparison failed to show any statistical differences. No differences were seen for brain weight in any dementia comparison. Abbreviations: AD: Alzheimer’s disease; HD: Huntington’s disease; PDD: Parkinson’s disease dementia; PMD: Post-mortem delay; VaD: Vascular dementia. | | | | |

| **Table S6.** Sensitivity analyses for urea post-mortem brain values | | | | | | |
| --- | --- | --- | --- | --- | --- | --- |
| Brain region | Control | VaD | *p*-value | RR | E-value | Glass’s Δ |
| Hippocampus | 12.49 | 29.41 | 0.047 | 2.4 | 4.23 | 4.01 |
| Cingulate gyrus | 15.49 | 35.33 | 0.041 | 1.82 | 3.04 | 3.34 |
| Frontal gyrus | 16.64 | 33.25 | 0.065 | 1.82 | 3.04 | 2.73 |
| Occipital cortex | 19.44 | 43.76 | 0.042 | 1.82 | 3.04 | 3.19 |
| Middle temporal gyrus | 19.98 | 43.63 | 0.046 | 1.82 | 3.04 | 3.32 |
| Basal ganglia | 16.31 | 38.40 | 0.035 | 2.73 | 4.9 | 4.12 |
| Thalamus | 14.94 | 33.85 | 0.045 | 1.36 | 2.03 | 3.69 |
| Abbreviations: RR: Risk ratio. | | | | | | |

**Supplementary Figures**

**Figure S1. Multiregional urea concentrations in four brain regions compared between control (red) and T2D (green) post-mortem tissue.** Data are means ± 95% CI. Five samples in the MN analysis could not be used due to a loss of analytes during the sample prep. All difference were non-significant. Non-standard abbreviations: FC: frontal cortex; HP: hippocampus; MN: meninges; TC: temporal cortex; T2D: type 2 diabetes.
